# Distinct Iron Deposition Profiles of Liver Zones in Various Models with Iron Homeostasis Disorders

**DOI:** 10.1002/advs.201800866

**Published:** 2018-10-12

**Authors:** Haoyang Song, Shuping Zhang, Xia Sun, Jing Liu, Yakun Wu, Wenli Guo, Fudi Wang, Xiaojuan Ou, Min Cong, Erhu Jin, Wenyong Li, Sijin Liu

**Affiliations:** ^1^ Anhui Province Key Laboratory of Embryo Development and Reproductive Regulation Anhui Province Key Laboratory of Environmental Hormone and Reproduction Fuyang Normal University Fuyang 236037 China; ^2^ State Key Laboratory of Environmental Chemistry and Ecotoxicology Research Center for Eco‐Environmental Sciences Chinese Academy of Sciences Beijing 100085 China; ^3^ Institute for Medical Engineering and Science Massachusetts Institute of Technology Cambridge MA 02139 USA; ^4^ Radiology Department Beijing Friendship Hospital Capital Medical University Beijing 100050 China; ^5^ University of Chinese Academy of Sciences Beijing 100049 China; ^6^ College of Fisheries Henan Normal University Xinxiang 453007 China; ^7^ QIMR Berghofer Medical Research Institute Brisbane 4029 Australia; ^8^ Department of Nutrition Nutrition Discovery Innovation Center Institute of Nutrition and Food Safety School of Public Health School of Medicine Zhejiang University Hangzhou 310085 China; ^9^ Liver Research Center Beijing Friendship Hospital Capital Medical University Beijing 100050 China

**Keywords:** iron deficiency, iron deposition, iron homeostasis, iron overload, liver zones

## Abstract

Determination of iron accumulation is crucial in diagnosing the occurrence and progression of many liver‐ and iron‐related diseases. Thus far, little is known about the profiles of iron deposition in different liver zones, particularly under conditions with disordered iron homeostasis. Here, uneven iron distribution in livers of patients with hereditary hemochromatosis (HH) is uncovered, showing the region with the highest iron concentration near the entrance site of the portal vein and hepatic artery in contrast to the sites with the lowest iron concentration close to the distal edge. Distinct iron distribution profiles are also found throughout liver zones in wild‐type mice and various mouse models with iron metabolism disorders, including hemochromatosis *(Hfe^−/−^*), iron deficiency, and inflammation. Of note, similar findings observed in HH patients are further demonstrated in *Hfe^−/−^* mice. Moreover, the zones with greater iron accumulation appear to be more sensitive to iron changes, e.g., there is iron increase upon iron overload and iron loss in response to iron deficiency. Mechanistic investigation manifests that these differential iron changes in liver zones are subjected to the regulation by the hepcidin–ferroportin axis. Additionally, the data corroborate the reliability of magnetic resonance imaging (MRI) in recognizing the differential iron deposition profiles among liver zones.

Clinical records have revealed that iron deposition is a common pathological feature of many liver diseases,^1^, ^2^ and animal studies have also demonstrated that hepatic iron deposition exacerbates the progression of liver diseases, such as fibrosis, cirrhosis, and even the occurrence of cancers.^3^, ^4^ Meanwhile, hepatic iron deposition was reported to impair the efficacy of interferon in antihepatitis.^5^ Thus, accurate examination of liver iron concentration (LIC) is crucial for the diagnosis of liver iron accumulation and liver‐related diseases. Considering the current assays, measurement of iron level in blood, e.g., serum ferritin concentration and transferrin‐iron saturation, cannot provide the direct and accurate LIC.^6^ Moreover, due to the inherent limitations, liver biopsy, as the dominant clinical examination strategy, suffers from such drawbacks as invasiveness, the requirements of sophisticated skills, variation, and poor reproducibility.^7^, ^8^, ^9^ Under this context, imaging has been becoming a promising tool for iron deposition examination, providing noninvasive, convenient, and reproducible visualization of hepatic iron deposition. To this end, multidetector computed tomography,^10^ magnetic resonance imaging (MRI),^11^, ^12^ and superconducting quantum interference device^13^, ^14^ have been being developed in identification and evaluation of iron deposition in the liver, offering an alternative noninvasive method for liver iron quantification in patients.

More and more studies demonstrate that liver is not a homogenous organ with distinct architecture and zonation. For example, hepatocytes along the lobules vary in differentiation state and metabolic regulatory machineries that are optimized for different hepatic functions.^15^, ^16^ Nonetheless, little is known on iron distribution along the lobules thus far, although iron homeostasis per se essentially dictates cell differentiation and priming state of hepatocytes.^17^, ^18^ Yet, mounting evidence suggests that iron is not evenly distributed in different parts of the liver, as reflected by varied LICs in different parts in animals.^19^, ^20^ But, no specific rule for iron distribution among zones have been reported, particularly under disordered iron homeostasis to date. Moreover, the clinical relevance and implications of such a rule have not been tested. Under this setting, more efforts are warranted to shed light on the iron accumulation profiles in different models, and to look into the diagnostic significance of differential iron deposition in diverse liver diseases. Thus, the objective of the current study is to seek the liver iron accumulation profiles among zones in various models with different iron homeostasis disorders.

First, we examined the hepatic iron distribution in patients with hereditary hemochromatosis (HH) using MRI. Five regions were randomly selected to determine the *R*_2_* values (**Figure** **1**A). Compared to the healthy individuals, LIC in each region, as reflected by the *R*_2_* value, was much higher in HH patients (Figure 1B). Considering the healthy individuals, the *R*_2_* value among five regions was nearly comparable to each other (Figure 1B). However, the pattern was dramatically changed in HH patients, with ≈1.4–1.8 fold increase in the *R*_2_* value at the 1 region of interest (ROI) (ROI‐1, with the highest LIC near the entrance site of the portal vein and hepatic artery) in comparison to the site at ROI‐5 (in other words, with the lowest LIC close to the distal end region of the liver) (Figure 1B). Although the *R*_2_* value varied among HH patients, a similar pattern was observed in all 5 HH patients, suggesting that hepatic iron is not evenly distributed in the liver in HH patients. Due to enhanced liver iron accumulation, significant serum alanine aminotransferase (ALT) and aspartate aminotransferase (AST) concentrations were found in HH patients compared to healthy control (Figure S1, Supporting Information, *P* < 0.001), indicative of hepatic injuries.^21^ When iron is overloaded in the liver, excessive iron either in transferrin‐bound form or nontransferrin‐bound form (namely nontransferrin‐bound iron) is accumulated in hepatocytes, causing cell death, collapse of hepatic lobular scaffold, and cirrhosis.^22^ Taking into account the current findings on the uneven distribution of iron across liver zones, it would be argued that differential extent of disease progression among these liver zones may be observed under iron overload disorders, which warrants further detailed investigation. Moreover, previous studies also suggested that chronic iron overload contributes to the development of hypertension and ventricular hypertrophy through inducing vascular dysfunction.^23^ Although the relationship between iron overload and hypertension remains intertwined, one would expect to see deteriorated zone‐specific iron deposition responding to portal hypertension.^24^ More efforts are necessary to shed light on this complex relationship.

**Figure 1:**
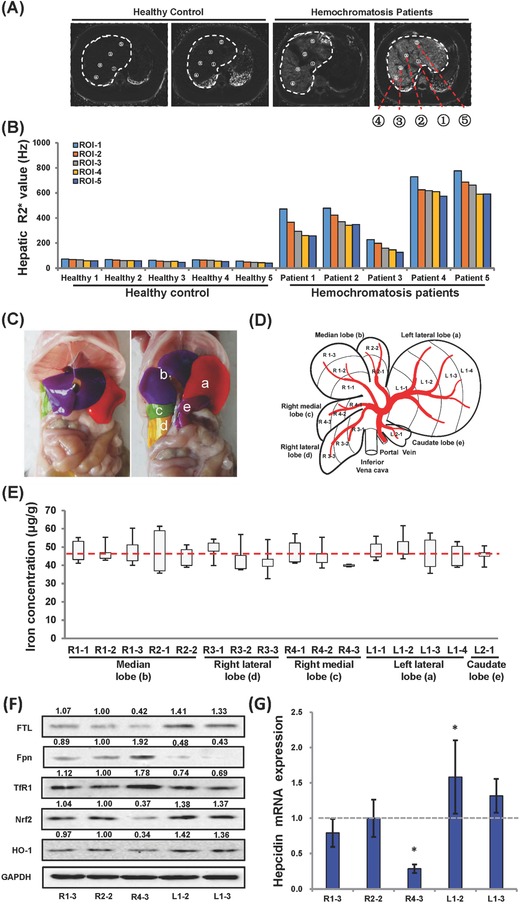
Hepatic iron distribution in humans and animals. A) The representative *R*_2_* images for LIC assessment in the healthy individuals and HH patients. B) Quantification of *R*_2_* relaxometry indicative of iron content. Points 1–5, randomly selected sites with differential distance to the entrance domain of the portal vein and hepatic artery. C) Photographs with intact illustration of mouse liver lobes (left) and unfolded illustration of mouse liver lobes (right). D) A schematic delineating the liver lobe arrangement and blood flow across the lobes. E) Hepatic iron mass in different zones in 8 weeks old *Wt* mice (*n* = 10). F) Protein content of light ferritin (FTL), Fpn, TfR1, Nrf2, and HO‐1, and G) hepcidin mRNA expression levels in different zones in *Wt* mice (*n* = 4–6). Hepcidin level in R2‐2 was defined as 1. Asterisk (*) indicates *P* < 0.05, compared to R2‐2.

To verify the above findings observed in HH patients, we next investigated the LIC in wild‐type (*Wt*) mice and various mouse models with iron disorders. The blood supply was spread from the entrance domain of the portal vein and hepatic artery to the distal end throughout the liver in mammals.^25^, ^26^ Given the architectural differences of liver lobes between human and mouse,^27^, ^28^ the murine five lobes were named from A to E with artificial colors, as depicted in Figure 1C. Taking into account the direction of blood flow, as delineated in Figure 1D, we deliberately divided the lobes into different zones based on the distance to the entrance domain of the portal vein and hepatic artery, with a comparable weight for each zone within the same lobe. Afterward, we individually determined LICs in 16 different zones. Strikingly, iron contents varied among different zones within each lobe, although no very convincing common rule could be defined for all lobes when considered individually (Figure 1E); however, there was an overall trend of gradient decrease of LIC from zones near the entrance site of the portal vein and hepatic artery to the distal end region of the liver. LIC variations were further confirmed by the levels of ferritin, the iron storage protein, in five representative zones, with the highest level in L1‐2 and the lowest level in R4‐3 (Figure 1F and Figure S2A, Supporting Information).

Hepcidin‐ferroportin (Fpn) axis is the fundamental signaling in governing iron homeostasis under physiological and pathological conditions.^29^ Hepcidin is expressed by hepatocytes to induce the degradation of Fpn, the solely known iron exporter in mammals.^30^ Hepcidin expression is concertedly regulated by a fine‐tuned machinery, where the basic level of hepcidin is controlled by iron concentration through bone marrow protein (BMP) signaling.^31^, ^32^ To test whether differentiated LICs would give rise to varied hepcidin levels in different zones, hepcidin expression was assessed through quantitative RT‐PCR (RT‐qPCR). As shown in Figure 1G, the hepcidin messenger RNA (mRNA) level was elevated approximately by 50% and 30% in L1‐2 and L1‐3, respectively, in contrast to ≈70% reduction in R4‐3, compared to that in R2‐2 (Figure 1G, *P* < 0.05), in agreement with the corresponding LIC in each zone. Subjected to the differential levels of hepcidin, the Fpn protein mass in each zone reversely correlated to their hepcidin levels (Figure 1F and Figure S2B, Supporting Information), highlighting a local regulation of hepcidin on Fpn.^33^ Thus, these results together unearthed the uneven iron distribution pattern in the liver of *Wt* mice, and also signified the vital role of hepcidin‐Fpn axis in dictating microenvironmental hepatic iron. We further looked into the mass of transferrin receptor (TfR1) in these zones. Subjected to the regulation by the intracellular iron level through the iron responsive element (IRE)‐iron responsive protein (IRP) mechanism, TfR1 mRNA stability would be compromised in response to replete cellular iron, giving rise to reduced protein mass, and TfR1 mass would be reversely elevated upon iron deficiency.^34^ Due to distinct iron levels in different zones, we observed differential TfR1 content in these zones, as a higher level of TfR1 content in R4‐3 and a lower level in L1‐2 and L1‐3 were observed relative to other zones, as demonstrated by Western blotting (Figure 1F and Figure S2C, Supporting Information). Responding to enhanced iron accumulation, elevated oxidative stress would be expected to occur due to the reactivity of iron in generating free radicals.^35^, ^36^ Therefore, we measured the malondialdehyde (MDA) levels. As shown in Figure S3 in the Supporting Information, a higher level in L1‐2 and L1‐3 and a lower level in L4‐3 for the tissue MDA were found, respectively, in agreement with their differential tissue iron content. In support of this finding, discrepant induction of crucial regulators against oxidative stress, including nuclear factor (erythroid‐derived2)‐like2 (Nrf2)^37^ and heme oxygenase‐1 (HO‐1),^38^ was demonstrated in these zones (Figure 1F and Figure S2D,E, Supporting Information), consistent with their corresponding iron levels.

To corroborate the above findings, we further employed various mouse models with disordered iron homeostasis. Hemochromatosis (*Hfe*) gene is a most important regulator of hepcidin expression, and genetic *Hfe* mutations represent a common mechanism for HH.^39^ Since we observed the biased iron distribution in patients with HH (Figure 1A,B), we further looked into iron distribution pattern in *Hfe^−/−^* mice. As shown in **Figure** **2**A, the average of LIC in *Hfe^−/−^* mice was much higher than that in *Wt* mice at different ages, consistent with the previous report.^40^ Similar to the findings in HH patients (Figure 1A,B), there was on overall trend of gradual drop of hepatic iron content from the entrance region to the edge region (Figure S4, Supporting Information). Moreover, analogous to *Wt* mice as observed in Figure 1E, LIC variations were also demonstrated in different zones in *Hfe^−/−^* mice from 4 to 12 weeks (Figure 2A and Figure S5A–C, Supporting Information). To substantiate the LIC variations, Prussian iron staining was carried out in sections from five representative zones of mice with different ages. In agreement with the LIC measurement (Figure 2A), iron accumulation appeared more pronounced in L1‐2 and L1‐3 zones than other zones, and the least iron accumulation was found in the R4‐3 zone in *Hfe^−/−^* mice at all tested ages (Figure 2B). Afterward, hepcidin expression was assayed in *Hfe^−/−^* mice. In line with previous reports,^41^ the hepcidin level was lower in *Hfe^−/−^* mice at younger ages (namely 4 and 8 weeks old) relative to that in *Wt* mice (Figure 2C, *P* < 0.05), and the hepcidin level rose a comparable level to that in *Wt* mice when *Hfe^−/−^* mice became older (12 weeks), due to sustained stimulation on hepcidin expression by mounting iron.^42^ Nonetheless, higher levels of hepcidin expression was demonstrated in L1‐2 and L1‐3 zones than other zones in *Hfe^−/−^* mice across all ages (Figure 2C, *P* < 0.05), in parallel to the observation in *Wt* mice (Figures 1G and 2C).

**Figure 2:**
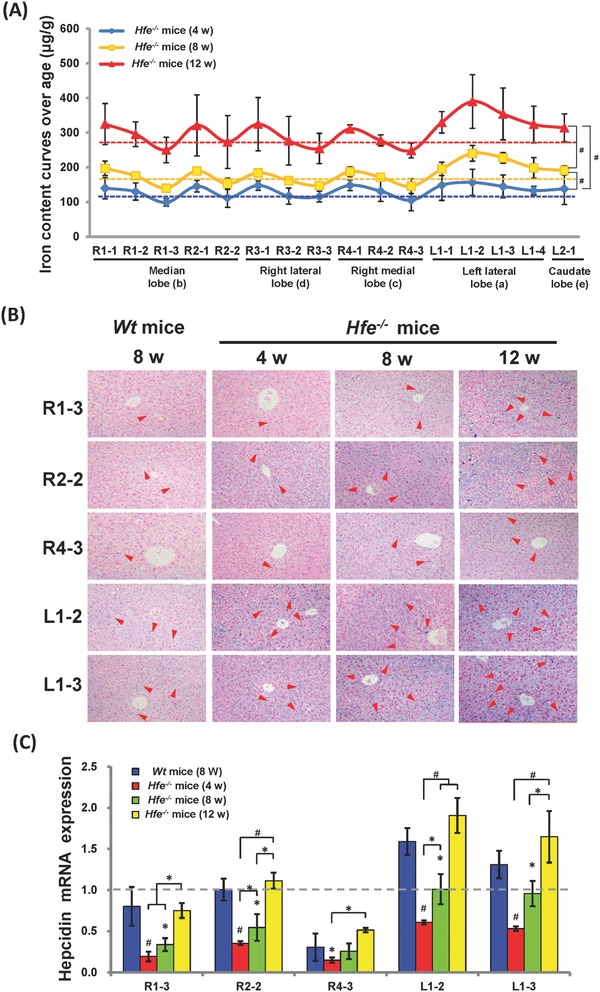
Hepatic iron distribution in *Hfe^−/−^* mice. A) Hepatic iron content curves of *Hfe^−/−^* mice over age (*n* = 10), and B) liver iron staining. Original magnification, ×200. Arrows point at iron accumulation. C) Hepcidin mRNA expression level in livers from *Wt* and *Hfe^−/−^* mice (*n* = 4–6). Hepcidin level in R2‐2 zone was set as 1. Asterisk (*) indicates *P* < 0.05 and pound (#) shows *P* < 0.001, relative to *Wt* control or as indicated.

Next, the net increased mass of LICs was calculated for each zone in *Hfe^−/−^* mice over age. As shown in Figure S5D in the Supporting Information, the ∆LICs were much greater in the left lateral lobe than other lobes for *Hfe^−/−^* mice from 4 to 8 weeks, suggesting a distinct capability of different zones in response to iron changes. The increase became similar from 8 to 12 weeks (Figure S5D, Supporting Information), as iron overload became more severe at older ages.^41^ Afterward, we employed additional mouse models: iron deficiency and acute systemic inflammation. Consistently, iron reduction in iron‐deficient mice relative to *Wt* mice were not even in each lobe, with smaller changes in the right lateral and medial lobes in contrast to greater changes in other lobes (Figure S6A,B, Supporting Information). Furthermore, we established a mouse model of acute systemic inflammation induced by lipopolysaccharides (LPS), as characterized by massive elevation of white blood cells and interleukin 6 (IL‐6) level in peripheral blood (Figure S7A,B, Supporting Information). In addition to stimulation on hepcidin expression by iron/BMP signaling, inflammation is also a robust driving force to promote hepcidin expression through IL‐6/ Interleukin 6 receptor signaling.^43^ As a result of IL‐6 induction, as shown in Figure S7C in the Supporting Information, hepcidin expression was promoted quickly by LPS after 6 h in all selected zones, especially in the L1‐1 and L1‐3 zones with more than twofold increase, compared to the untreated control (*P* < 0.001), in analogy to the induction of serum IL‐6 (Figure S7B, Supporting Information). By contrast, no significant induction of hepcidin was found in R4‐3 zone (Figure S7C, Supporting Information). Moreover, hepcidin expression chronobiologically responded to IL‐6 changes with a drop over time (Figure S7C, Supporting Information), indicating a great correlation between hepcidin induction and IL‐6 stimulation. Due to hepcidin induction, iron egress out of macrophages will be repressed, leading to elevation of iron content in organs.^44^ As a consequence, we observed increased LICs in all zones from mice 48 h after LPS administration, compared to untreated control, and a greater increase was found in L1‐2 and L1‐3 zones relative to other zones (Figure S7D, Supporting Information), in agreement with the changes of IL‐6 levels. These data further pinpointed the distinct capability of different zones responding to exotic signaling molecules to mobilize iron coming‐in and going‐out.

In the following, we endeavored to corroborate the above findings on distinct iron accumulation in different lobes and zones and their differential capability to mobilize iron ingress and egress. To reach this aim, we used ^57^Fe as the tracer to determine iron changes in each zone in *Wt* mice and *Hfe^−/−^* mice. As shown in **Figure** **3**A, ^57^Fe uptake was unevenly distributed among the lobes, with the least content in the right medial lobe and an overall higher level in the left lateral lobe, consistent with the LIC results as described in Figure 1E. Moreover, ^57^Fe mass was also found differentially deposited in different zones within each lobe, consistent with the LIC measurement results (Figure 1E). Additionally, *Hfe^−/−^* mice exhibited a similar distribution pattern of ^57^Fe uptake to *Wt* mice (Figure 3A,B), and also showed a constant pattern to that of LIC data (Figure 2A and Figure S5A–C, Supporting Information). Therefore, these ^57^Fe data substantiated varied uptake and deposition of iron in different lobes and zones.

**Figure 3:**
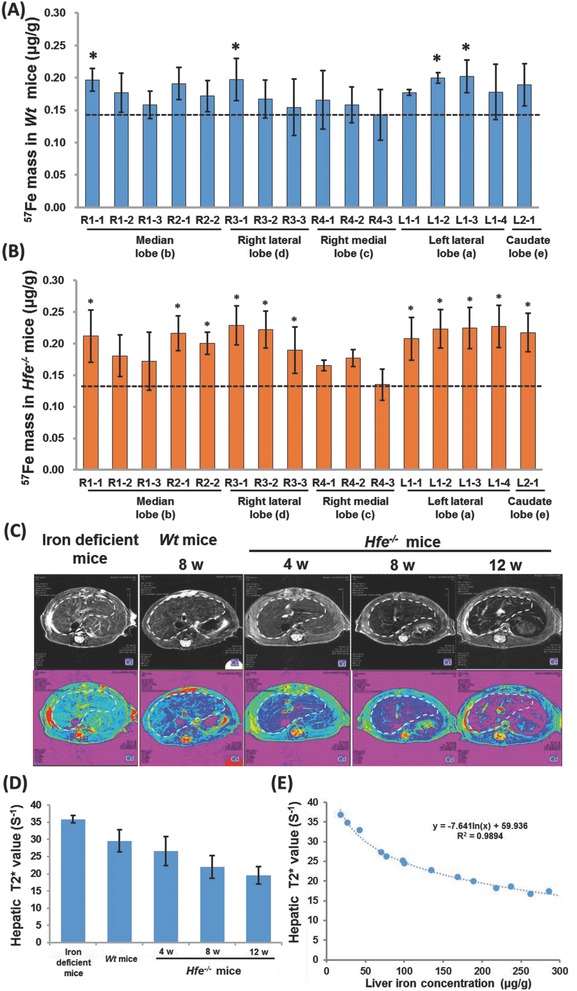
^57^Fe uptake assessment and quantitative MRI determination. A,B) Hepatic ^57^Fe content in different liver zones in 8 weeks old *Wt* mice and *Hfe^−/−^* mice. Asterisk (*) indicates *P* < 0.05, relative to that in R4‐3 zone. C) Representative images of MRI *T*_2_* (upper) and *T*_2_* pseudo‐color mapping (lower) of mouse livers. D) Quantified *T*_2_* values of selected ROIs (5 mm^2^) at the constant sites for iron‐deficient mice, *Wt* mice, and *Hfe^−/−^* mice with different ages. The sites were selected in the right lobe of the liver by avoiding the locations of lobe edges and the apparent artificial shadow of large blood vessels and bile ducts. E) The correlation analysis between hepatic *T*_2_* and LIC.

Finally, to explore the implications of the current study in bridging biochemical measurement to imaging, we used MRI to recognize and quantify hepatic iron. Along with the increase of LICs from iron‐deficient mice, to *Wt* mice and *Hfe^−/−^* mice at different ages, the color of the MRI *T*_2_* pseudo‐color profile, representative of iron signal, gradually changed from blue to pink (Figure 3C), revealing mounting iron deposition. Specifically, in iron‐deficient mice, only light blue spots appeared around the entrance site of the portal vein and hepatic artery, in contrast to enlarged blue area in the pseudo‐color map in *Wt* mice. Differently, livers from *Hfe^−/−^* mice displayed gradual enhancement of red/pink signal over age (Figure 3C). Importantly, the Fe color was observed to spread out from the entrance area of the portal vein and hepatic artery to the distal end in *Hfe^−/−^* mice (Figure 3C). Quantified data showed a constant decline of *T*_2_* value from iron‐deficient mice, to *Wt* mice and *Hfe^−/−^* mice over age (Figure 3D), depicting a constant increase of iron accumulation in these mice. Further analysis manifested a close correlation between *T*_2_* values and LIC measurements, verifying the reliability of MRI in determining the differential iron deposition signature in different zones (Figure 3E).

To summarize, in the current study, we recognized varied LICs in different regions of the livers with iron overload in HH patients, mainly characterized by the gradient decrease of LIC from regions near the entrance site of the portal vein and hepatic artery to the distal end region of the liver. We also uncovered the pattern of liver iron accumulation profiles in different lobe and zones using *Wt* and diverse mouse models of iron disorders. Of note, similar findings in HH patients were demonstrated in *Hfe^−/−^* mice. The underlying mechanisms were accounted for by the differential sensitivities among liver zones in response to iron level changes and exotic regulators under the regulation of the hepcidin‐Fpn axis. Moreover, we demonstrated the reliability of MRI in accurately determining such iron deposition profiles. This study unearthed the diagnostic significance of distinct hepatic iron distribution profiles in determining iron accumulation for iron disorders, and would open a new path to study iron‐associated risks for different liver zones under various diseases.

## Experimental Section

Clinical Data and R_2_* Measurement: MRI data on two HH patients and two age‐matched healthy individuals were obtained from Beijing Friendship Hospital with written informed consent form. *R*_2_* measurements were determined by collecting gradient echo multi‐echo MR images at increasing echo time as previously described.^11^, ^45^

*Animal Experimentation*: Wild‐type BABL/c mice and the *Hfe^−/−^* mice with 129S genetic background, a gift from Dr. Fudi Wang, were raised under a specific pathogen free (SPF) facility. To induce iron deficiency, four weeks old *Wt* mice after weaning were raised on low‐iron diet (4 ppm iron) for 3 weeks. To induce acute inflammation, mice were intraperitoneally injected with LPS (Sigma) at 100 µg/kg. Control mice received phosphate buffer saline (PBS) only. Mice were sacrificed at different time points after LPS administration, along with the collection of the peripheral blood and organs for further assays. The experimental protocols were approved by the Animal Ethics Committee of the Research Center for Eco‐Environmental Sciences, Chinese Academy of Sciences.

*RT‐qPCR, Western Blot, CBC, and ELISA Analyses*: Gene expression was determined by RT‐qPCR, and protein concentrations were assessed through Western blot analysis, as previously described in the laboratory.^46^, ^47^, ^48^ Primers and antibodies used here are listed in Table S1 and Table S2 in the Supporting Information, respectively. Glyceraldehyde‐3‐phosphate dehydrogenase (GAPDH) was used as internal control for normalization. Twenty microliters of collected peripheral blood was diluted (1:100) with a standard dilution buffer, followed by complete blood count (CBC) analysis on a hematology analyzer (Nihon Kohden). Serum IL‐6 levels were assayed using a commercial ELISA kit from (OriGene, USA) following the instructions provided by the manufacturers.

*Hepatic MDA Assay*: Hepatic MDA content was assayed using a kit purchased from the Nanjing Jiancheng Bio‐Engineering Research Institute Co., Ltd. (Nanjing, China), following the instructions provided by the manufacturer.

*Tissue Iron Assessment and Histological Examination*: Hepatic nonheme iron mass was assessed, as previously described.^33^ Histological examination of tissue specimens was performed with hematoxylin and eosin (H&E) according to the standard protocol, and iron staining was carried out using the method of Prussian blue stain, as descried.^33^

*^57^Fe Uptake Determination*: Stable Fe isotope ^57^Fe (^57^Fe at 94%; Frontier Scientific Inc., USA) was used as the tracer to look at iron uptake. In brief, ^57^FeCl_3_ solution (at 0.4 mol L^−1^) was prepared through dissolution of 22.85 g ^57^FeCl_3_ in HCl overnight, and was then stored at 4 °C. ^57^FeCl_3_ solution and the vehicle solution were adjusted to pH 7 prior to administration. Eight weeks old mice were fasted overnight, and were then orally administrated with 20 µL of ^57^FeCl_3_ or vehicle solution. After 8 h, mice were sacrificed, followed by liver specimen collections. Thereafter, ^57^Fe mass in liver tissue were quantified through inductively coupled plasma mass spectrometry analysis, as previously reported.^49^

*MRI Assessment in Animals*: All MRI assessments for iron deposition on mice were performed on a 9.4T Bruker Biospec scanner (Bruker Biospin, Germany) at the National Center for Nanoscience and Technology, Chinese Academy of Sciences, following the method as reported previously.^50^, ^51^, ^52^ Determination of the signals on the acquired images was carried out using MATLAB (Mathworks Inc., MA). For individual image, ROIs on the same layer were selected, and then *T*_2_* value was measured pixel by pixel using a routine least‐squares fitting algorithm.

*Quantitative Analysis of Autoradiograms*: The intensity of autoradiogram for each lane was quantified with the software Image J (NIH, http://rsbweb.nih.gov). The quantified data of target proteins were normalized to those of the loading control, GAPDH, for each lane.

*Statistical Analysis*: Independent *t*‐test, paired *t*‐test, and one‐way ANOVA test were accordingly employed to analyze experimental data. Data were shown in mean ± SD. *P* < 0.05 was considered statistically significant.

## Conflict of Interest

The authors declare no conflict of interest.

## Supporting information

Supplementary
