# Supplementary material for: Distinct Iron Deposition Profiles of Liver Zones in Various Models with Iron Homeostasis Disorders
Source: Adv Sci (Weinh). 2018 Oct 12;5(11):1800866. doi: 10.1002/advs.201800866 (PMC6247051; doi:10.1002/advs.201800866)
Supplement: Supplementary file 1 — Supplementary [file ADVS-5-1800866-s001.pdf]

Copyright WILEY-VCH Verlag GmbH & Co. KGaA, 69469 Weinheim, Germany, 2018.

ADVANCED SCIENCE
Open Access

Supporting Information

for *Adv. Sci.,* DOI: 10.1002/advs.201800866

Distinct Iron Deposition Profiles of Liver Zones in Various Models with Iron Homeostasis Disorders

Haoyang Song, Shuping Zhang, Xia Sun, Jing Liu, Yakun Wu, Wenli Guo, Fudi Wang, Xiaojuan Ou, Min Cong, Erhu Jin, Wenyong Li, and Sijin Liu**

Supplementary Information for Online Publication

Supplementary Tables

Table S1. The primers for RT-qPCR assay.

Gene symbol	Sequence
GAPDH	F：5'-AAGGTCATCCCAGAGCTG-3’
	R：5'-GCCATGAGGTCCACCACCCT-3’
Hepcidin	F：5'-CTGAGCAGCACCACCTATCTC-3'
	R：5'-TGGCTCTAGGCTATGTTTTGC-3’

Table S2. Antibodies for Western blot analysis.

Antibody	Manufacturer	Dilution
mouse monoclonal anti-GAPDH	Santa Cruz Biotechnology	1: 1000
rabbit polyclonal anti-mouse Fpn1	Proteintech	1:2000
rabbit polyclonal anti-FTL	Proteintech	1:2000
rabbit polyclonal anti-TfR1	Proteintech	1:2000
rabbit polyclonal anti-Nrf2	Proteintech	1:2000
rabbit polyclonal anti-HO-1	Proteintech	1:2000
anti-mouse 2nd antibody	Proteintech	1:5000
anti-rabbit 2nd antibody	Proteintech	1:5000

Abbreviation list

HH	Hereditary hemochromatosis
Hfe$^{-/-}$	Including hemochromatosis
MRI	Magnetic resonance imaging
LIC	Liver iron concentration
CT	Computed tomography
SQUID	Superconducting quantum interference device
ROI	Region of interest
ALT	Alanine aminotransferase
AST	Aspartate aminotransferase
NTBI	Namely non-transferrin bound iron
Wt	Wild type
Fpn	Ferroportin
BMP	Bone marrow protein
qRT-PCR	Quantitative RT-PCR
TfR1	Transferrin receptor 1
IRE	Iron responsive element
IRP	Iron responsive protein
MDA	Malondialdehyde
Nrf2	Nuclear factor (erythroid-derived2)-like2
HO-1	Heme oxygenase-1
Hfe	Hemochromatosis

LPS	Lipopolysaccharides
WBCs	White blood cells
IL-6	Interleukin 6
IL-6R	Interleukin 6 receptor
TE	Echo time
CBC	Complete blood count
H&E	Hematoxylin-eosin
ICP-MS	Inductively coupled plasma mass spectrometry
ROIs	Regions of interest
L-ferritin	light ferritin

Figure S1

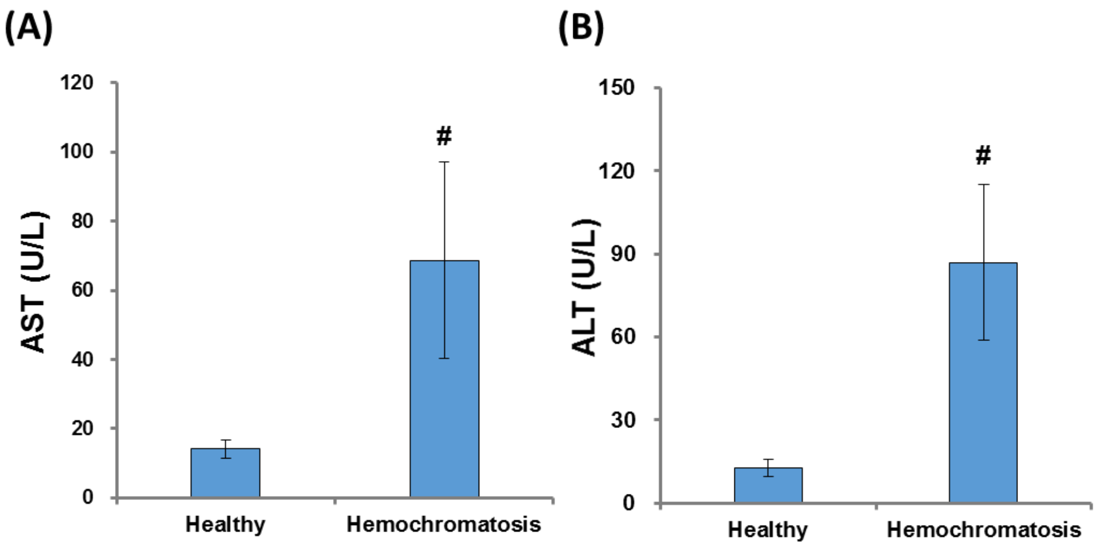

Figure S1. Serum concentrations of aspartate transaminase (AST) and alanine aminotransferase (ALT) in healthy individuals and HH patients.

Concentrations of (A) AST and (B) ALT in healthy individuals and HH patients (n=5).

Figure S2

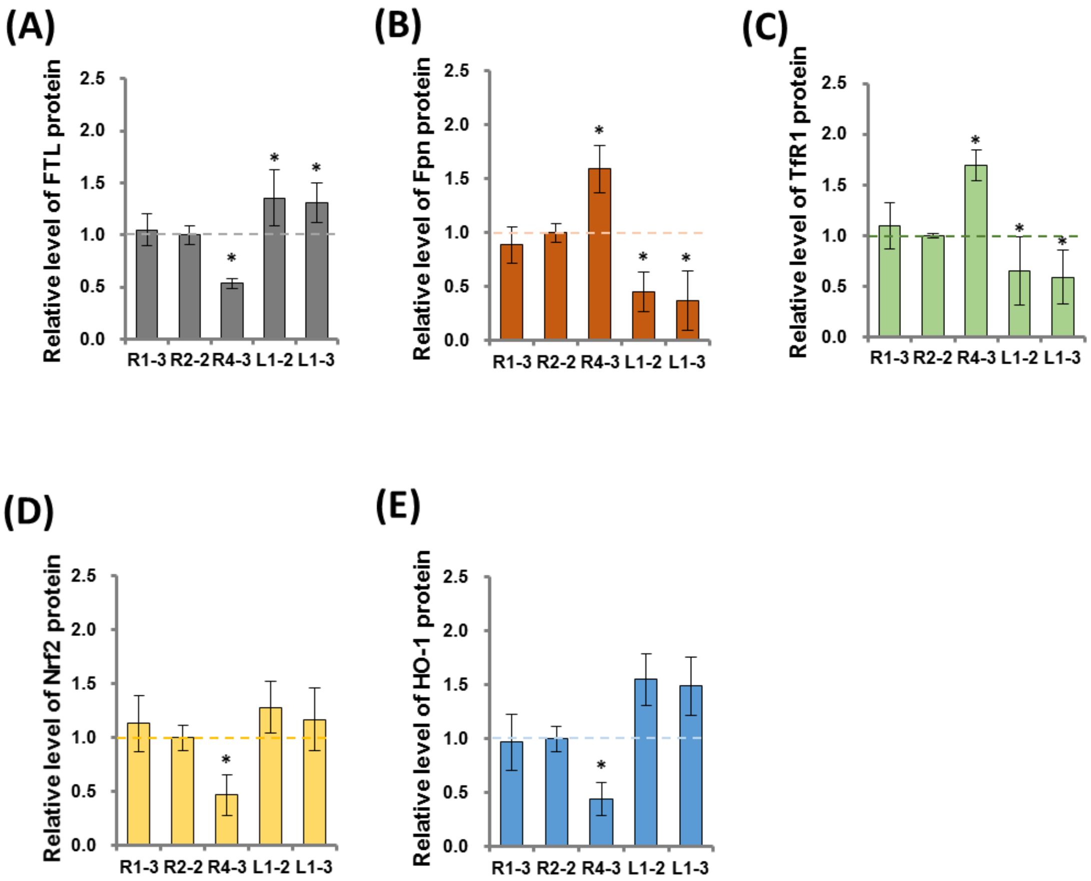

Figure S2. Quantified data for proteins in different zones of the livers from *Wt* mice.

(A-E) Quantification of protein concentrations of Fpn, FTL, TfR1, Nrf2 and HO-1 (n=5).

Figure S3

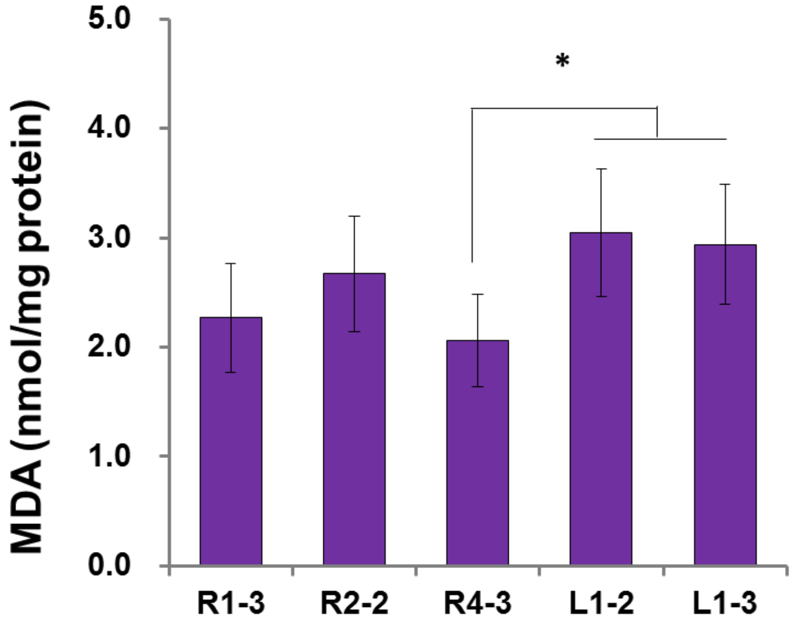

Figure S3. Hepatic MDA levels in different regions of the livers from *Wt* mice.

Content of MDA in different regions of the liver (n=5).

Figure S4

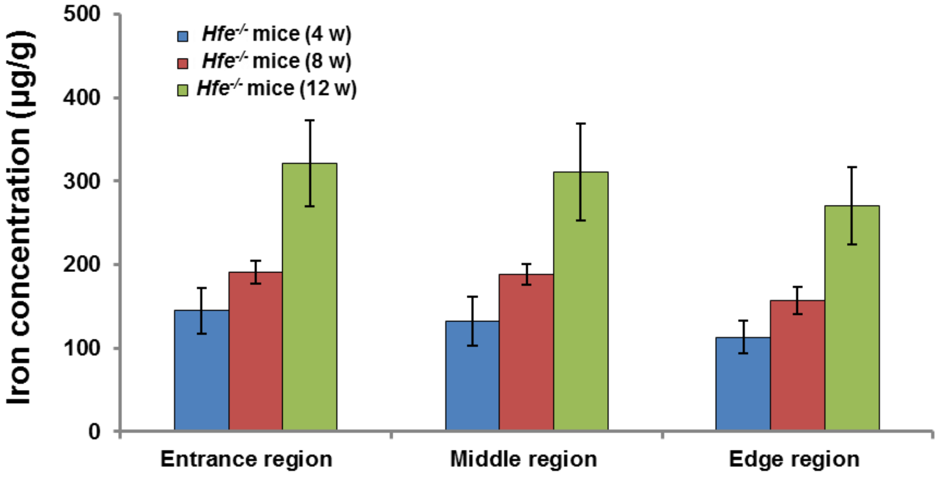

Figure S4. Iron deposition in different regions of the livers from *Hfe*$^{-/-}$ mice with different ages.

The liver was divided into three big regions based on the distance to the entrance site domain of the portal vein and hepatic artery, as entrance region (including R1-1, R2-1, R3-1, R4-1, L1-1, and L2-1), middle region (R1-2, R3-2, R4-2, L1-2 and L1-3) and edge region (R1-3, R2-2, R3-3, R4-3 and L1-4). Iron mass was determined for each region. There are 10 mice in each group (n = 10).

Figure S5

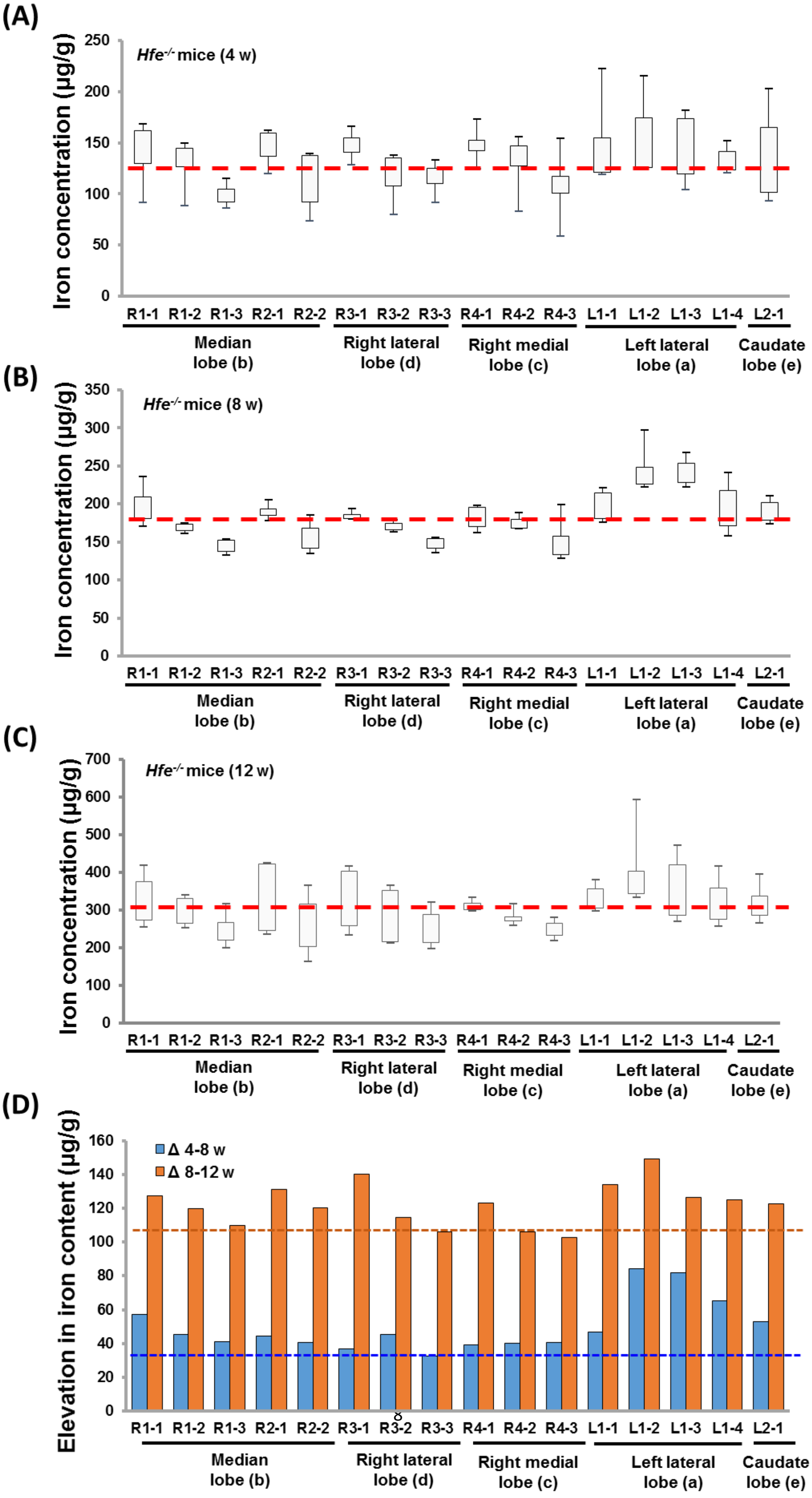
(A)
Hfe-/- mice (4 w)
Iron concentration (µg/g)
0
50
100
150
200
250
R1-1 R1-2 R1-3 R2-1 R2-2 R3-1 R3-2 R3-3 R4-1 R4-2 R4-3 L1-1 L1-2 L1-3 L1-4 L2-1
Median lobe (b)
Right lateral lobe (d)
Right medial lobe (c)
Left lateral lobe (a)
Caudate lobe (e)
(B)
Hfe-/- mice (8 w)
Iron concentration (µg/g)
0
50
100
150
200
250
300
350
R1-1 R1-2 R1-3 R2-1 R2-2 R3-1 R3-2 R3-3 R4-1 R4-2 R4-3 L1-1 L1-2 L1-3 L1-4 L2-1
Median lobe (b)
Right lateral lobe (d)
Right medial lobe (c)
Left lateral lobe (a)
Caudate lobe (e)
(C)
Hfe-/- mice (12 w)
Iron concentration (µg/g)
0
100
200
300
400
500
600
700
R1-1 R1-2 R1-3 R2-1 R2-2 R3-1 R3-2 R3-3 R4-1 R4-2 R4-3 L1-1 L1-2 L1-3 L1-4 L2-1
Median lobe (b)
Right lateral lobe (d)
Right medial lobe (c)
Left lateral lobe (a)
Caudate lobe (e)
(D)
Δ 4-8 w
Δ 8-12 w
Elevation in iron content (µg/g)
0
20
40
60
80
100
120
140
160
R1-1 R1-2 R1-3 R2-1 R2-2 R3-1 R3-2 R3-3 R4-1 R4-2 R4-3 L1-1 L1-2 L1-3 L1-4 L2-1
Median lobe (b)
Right lateral lobe (d)
Right medial lobe (c)
Left lateral lobe (a)
Caudate lobe (e)

Figure S5. Hepatic iron distribution for *Hfe*$^{-/-}$ mice over age

Iron content in different liver zones for *Hfe*$^{-/-}$ mice at 4 weeks (A), 8 weeks (B) and 12 weeks (C). (D) Increase in iron content for each zone from 4 to 8 weeks (△4-8 W) and from 8 to 12 weeks (△8-12 W). There are 10 mice in each group for analysis (n = 10).

Figure S6

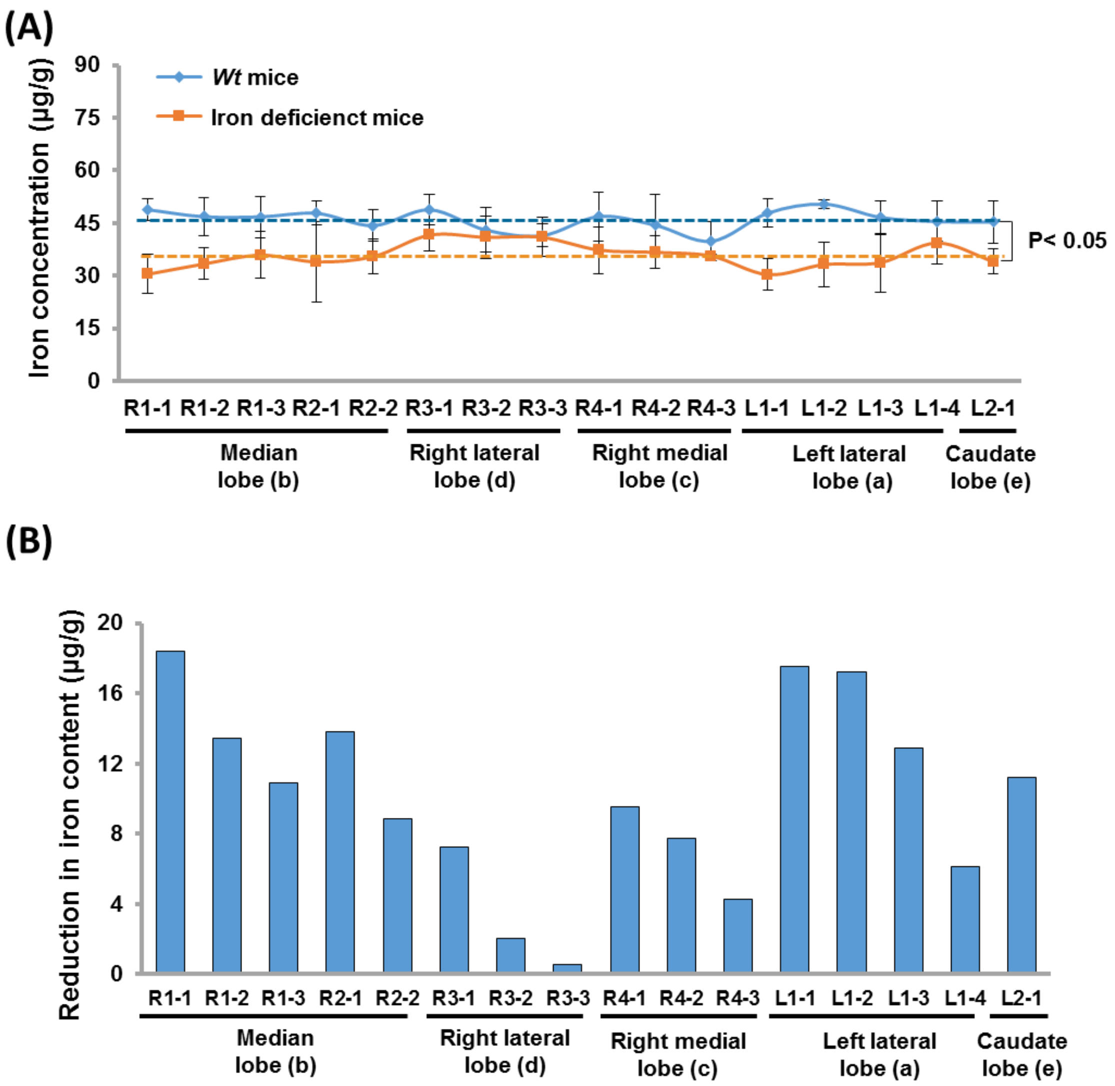

Figure S6. Hepatic iron distribution for iron deficient mice

(A) LICs and (B) reduction in iron content for each zone in iron deficient mice relative to WT mice (△iron change) for different liver zones in iron deficient mice in comparison to age-matched *Wt* mice. There are 10 mice in each group for analysis (n = 10).

Figure S7

(A)

	PBS	LPS (6 h)	LPS (24 h)	LPS (48 h)
WBC($\times 10^9$/L)	3.41 ± 0.54	3.12 ± 2.86	3.78 ± 1.19	5.23 ± 0.15
LY%	36.34 ± 4.15	80.42 ± 4.96 #	82.41 ± 18.72 #	84.13 ± 6.27 #
LY($\times 10^9$/L)	0.48 ± 0.19	2.64 ± 2.50	4.16 ± 0.92 *	6.97 ± 0.59 *
RBC ($\times 10^{12}$/L)	11.38 ± 0.41	10.84 ± 0.44	10.38 ± 0.43	10.08 ± 0.86
HGB (g/L)	164.78 ± 6.72	163.57 ± 9.61	157.78 ± 9.81	153.24 ± 11.53
HCT%	53.53 ± 2.32	50.76 ± 2.26	48.14 ± 2.55 *	49.27 ± 3.96
MCV (fL)	46.93 ± 0.51	46.74 ± 0.47	46.32 ± 1.24	46.83 ± 0.4
MCH (pg)	14.52 ± 1.06	15.28 ± 0.31	15.18 ± 0.36	15.24 ± 0.32
MCHC (g/L)	308 ± 23.62	322.6 ± 6.02	327.99 ± 11.6	310.67 ± 4.04

(B)

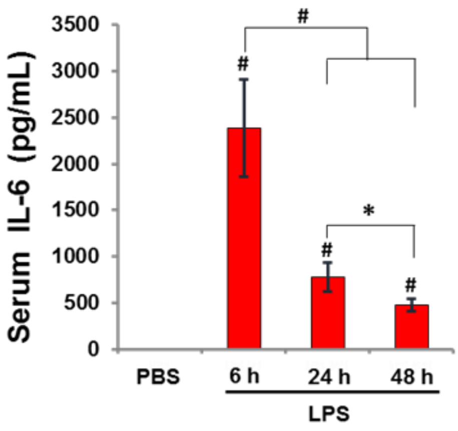

(C)

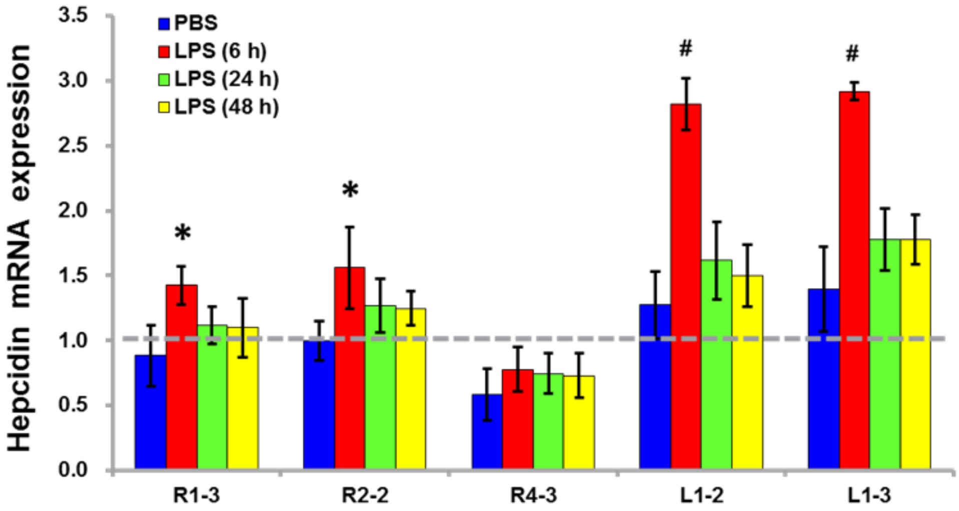

(D)

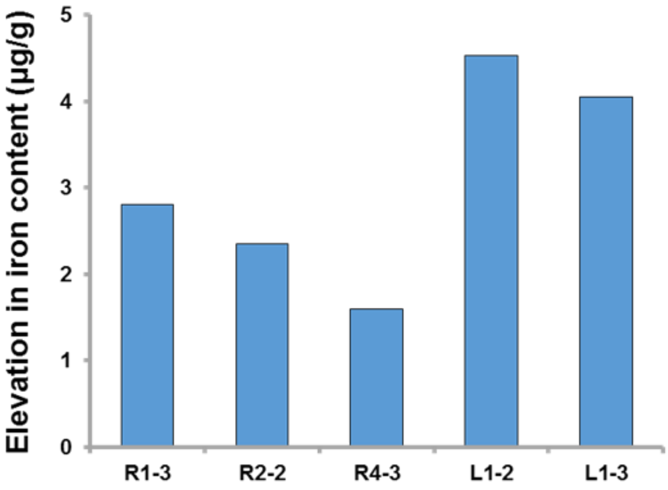

Figure S7. Hepatic iron changes for mice under inflammation

(A) The results of CBC analysis, (B) serum IL-6 concentrations and (C) hepcidin mRNA expression levels for *Wt* mice under inflammation over time. Mice were treated with LPS at 100 μg/kg body weight for 6, 24 and 48 h. Hepcidin expression level in R2-2 of untreated mice was defined as 1. (D) Elevation in iron content 48 h after LPS treatment relative to untreated control in five representative zones (△iron change). There are 10 mice in each group for analysis (n = 10). Asterisk (*) indicates $P < 0.05$, and pound (#) points at $P < 0.001$, compared to untreated mice.
